# Next generation tools for genomic data generation, distribution, and visualization

**DOI:** 10.1186/1471-2105-11-455

**Published:** 2010-09-09

**Authors:** David A Nix, Tonya L Di Sera, Brian K Dalley, Brett A Milash, Robert M Cundick, Kevin S Quinn, Samir J Courdy

**Affiliations:** 1 Department of Oncological Sciences, University of Utah, Huntsman Cancer Institute, Salt Lake City, USA; 2 Research Informatics, Huntsman Cancer Institute, Salt Lake City, USA; 3 Microarray/Next Generation Sequencing Shared Resource, Huntsman Cancer Institute, Salt Lake City, USA; 4 Bioinformatics Shared Resource, University of Utah, Huntsman Cancer Institute, Salt Lake City, USA

## Abstract

**Background:**

With the rapidly falling cost and availability of high throughput sequencing and microarray technologies, the bottleneck for effectively using genomic analysis in the laboratory and clinic is shifting to one of effectively managing, analyzing, and sharing genomic data.

**Results:**

Here we present three open-source, platform independent, software tools for generating, analyzing, distributing, and visualizing genomic data. These include a next generation sequencing/microarray LIMS and analysis project center (GNomEx); an application for annotating and programmatically distributing genomic data using the community vetted DAS/2 data exchange protocol (GenoPub); and a standalone Java Swing application (GWrap) that makes cutting edge command line analysis tools available to those who prefer graphical user interfaces. Both GNomEx and GenoPub use the rich client Flex/Flash web browser interface to interact with Java classes and a relational database on a remote server. Both employ a public-private user-group security model enabling controlled distribution of patient and unpublished data alongside public resources. As such, they function as genomic data repositories that can be accessed manually or programmatically through DAS/2-enabled client applications such as the Integrated Genome Browser.

**Conclusions:**

These tools have gained wide use in our core facilities, research laboratories and clinics and are freely available for non-profit use. See http://sourceforge.net/projects/gnomex/, http://sourceforge.net/projects/genoviz/, and http://sourceforge.net/projects/useq.

## 

The post-genomic era holds many promises for addressing fundamental questions regarding biology and improving patient outcome through personalized medicine. It also presents several unique challenges that need to be addressed to maximize the effectiveness of using genomic data in the laboratory and clinic. One key issue is the exponential growth in the number, size, and complexity of datasets generated from genomic experiments. The bottleneck is less the cost and difficulty of generating the data but, more so, efficiently managing, analyzing, and distributing it. Here, we present three, open source, platform independent, software tools that we have developed to address each of these issues. These include a genomic LIMS and analysis project center (GNomEx), a GUI for wrapping command line analysis tools (GWrap), and a web application for programmatically distributing genomic data (GenoPub) to DAS/2-enabled applications such as the Integrated Genome Browser [1]. These form the basis of a genomic data pipeline used in our core facilities and research laboratories where genomic data is generated from bio-specimens in a chain-of-custody type tracking, annotation and distribution system (figure 1).

**Figure 1:**
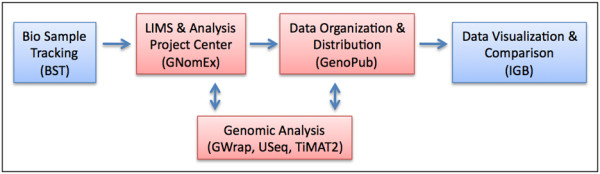
**Genomic data analysis pipeline**. Bio-specimens for genomic experimentation are submitted to the Microarray and Genomic Shared Resource (MGSR) using the GNomEx LIMS. If derived from patients, the samples are first annotated using the Bio Sample Tracking database. The MGSR uses GNomEx to process the samples through microarray or next generation sequencing experiments and to distribute the raw data. Genomic analysis is performed on the raw data and uploaded into GNomEx's analysis project center. The GWrap GUI command line wrapper is available for local processing using the USeq and TiMAT2 analysis packages. Final genomic analysis (e.g. track data) is annotated, organized, and distributed to DAS/2-enabled genome applications such as IGB using GenoPub. Red boxes denote applications discussed in this paper, blue boxes represent related applications detailed elsewhere.

## Application: GNomEx (Genomic Experiment Data Repository and Analysis Project Center)

### Background

GNomEx was developed to track samples for experimentation in our microarray and next generation sequencing core facility, associate raw data with biological samples, and link downstream computational analysis with the generated data. It is both a genomic LIMS and analysis project center designed for use by institutional core facilities and large research laboratories. Our installation of GNomEx [2] currently hosts ~7000 experiment requests, ~30,000 raw microarray and next generation sequencing datasets, and ~130 processed genomic analyses.

### Implementation and Results

#### 1) Web browser based interface

Adobe's open framework rich client Flex interface is used to provide a front-end graphical interface in one's preferred web browser using the Flash media player.

#### 2) Platform independent

Particular attention was made to achieve platform independence for all aspects of the software. These include a client-side Flex/Flash interface, Java programming language, an open source object-database mapping (Hibernate) that supports most relational databases (e.g. MySQL, Microsoft SQL Server, Oracle), and the deployment of the web-based applications using an open access J2EE application server (Orion). These choices allow other groups to install and use these applications within their existing infrastructure.

#### 3) Sample annotation

GNomEx is built around the concept of projects in which individual experiments are grouped. Users are encouraged, through a wizard-like interface, to associate annotations with their projects and experiments. Where appropriate, MGED ontologies [3], have been used to populate these annotation categories to assist in organizing, grouping, and searching of projects and experiments.

#### 4) Public-private access

Experiment annotations, data files, and associated data analysis files are safeguarded by a robust security manager that restricts access to authenticated users. The visibility of an experiment is set to either public, members, or members and collaborators. Following publication, researchers are encouraged to make their raw and analyzed data publicly available by changing their visibility settings. This will allow guest users to browse, search, and download published data.

#### 5) Experiment submission

**Figure 2:**
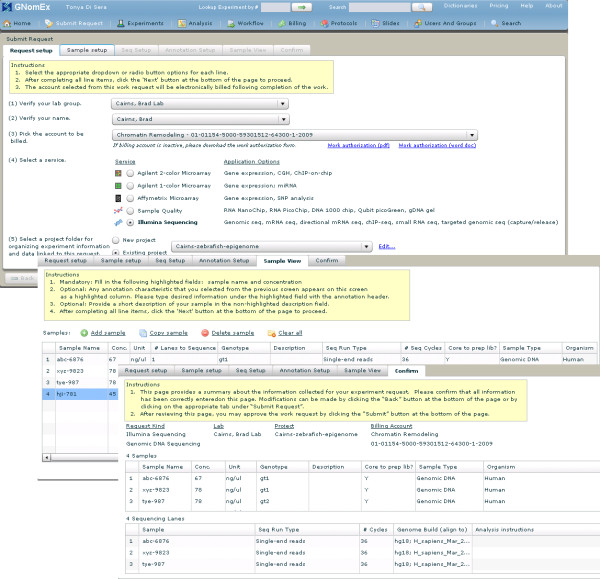
**Experiment submission**. Several screen capture images showing forms for submitting an experiment request to GNomEx. In this example, a request is being made for Illumina Sequencing with chIP-seq samples as well as secondary bioinformatic analysis.

Clients are stepped through the process of submitting an experiment using tabbed forms in a wizard-like fashion (figure 2). This includes selecting the account to be billed, the service to be performed (e.g. Agilent or Affymetrix microarray, sample quality, Illumina sequencing), and a project folder for organizing the experiment and associated raw data files. Pull-down menus are filtered based on prior selections driven by editable dictionaries, microarray/sequencing application parameters, and protocols. A series of screens are used to describe and annotate samples with MGED and user defined ontologies. The existing GNomEx services and forms are configured for Agilent and Affymetrix microarrays, Illumina sequencing, and Agilent Bioanalyzer quality control services. Reconfiguring these services to support alternative platforms and services is made possible with modifications to the dictionaries, slides, and protocol objects.

#### 6) Slides

**Figure 3:**
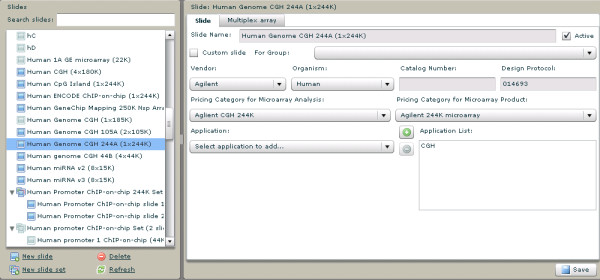
**Microarray slide tracking**. A screen capture showing information related to all of the microarrays in use at the University of Utah's Microarray Core facility. Options are available for adding single and multiplexed slides, specifying their design files, and associated applications.

Information relevant to microarray slides/chips is entered and tracked through a dedicated "Slides" interface (figure 3). Slide/chip name, catalog number, vendor, organism, application, multiplexing capability and pricing are recorded within this interface. This information is critical for guiding the client through selection of appropriate microarray products and for analysis of data files generated from microarray hybridizations.

#### 7) Laboratory workflows

**Figure 4:**
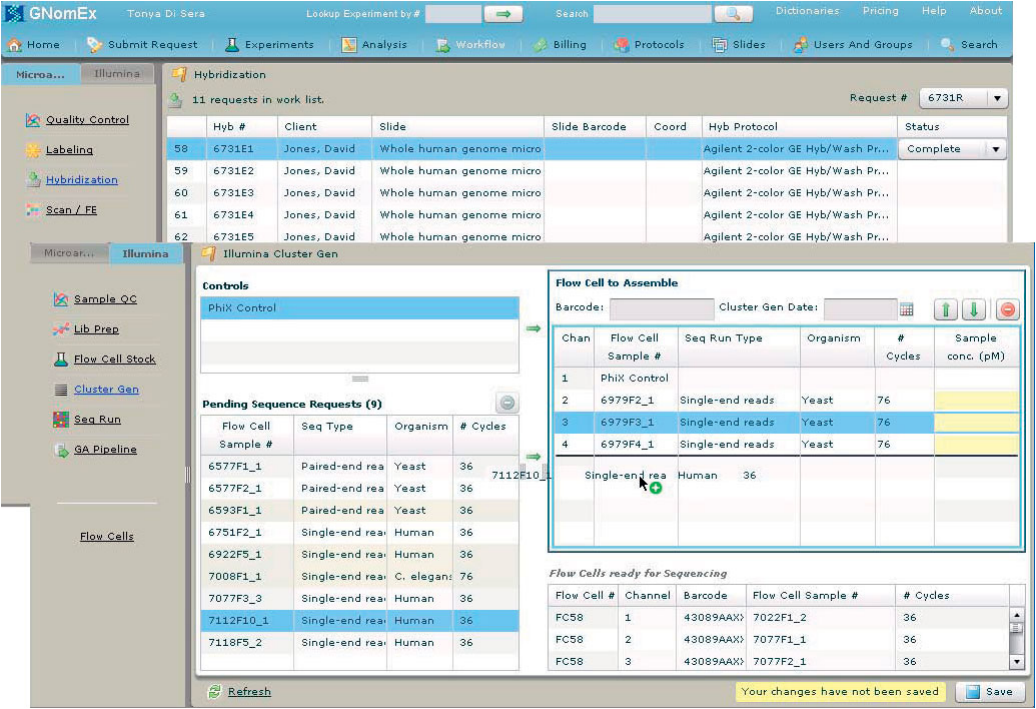
**Laboratory workflows**. Shown here are several screen captures of the microarray and sequencing workflows. These are used in the laboratory to track the processing of samples through each experiment, detail quality control metrics, and facilitate parallel processing of samples to increase efficiency. In these images, virtual flow cells are being assembled for sequencing.

GNomEx is built to manage and track experiment requests in the core facility through two customized sample processing workflows (figure 4). The Microarray workflow is designed to document information concerning sample quality labeling, microarray hybridization, scanning, and feature extraction. The Sequencing workflow is currently configured to support the Illumina Genome Analyzer GAIIx and HiSeq2000. It tracks sample quality, library preparation, cluster generation, sequencing, and GA Pipeline processing. Experimental details about each sample are recorded using work lists. Core facility personnel indicate completion of the step, track part numbers, record experimental parameters and document laboratory protocols. Automated e-mail functionality provides a communication link between the core facility and the client to indicate the completion of key steps in the experimental process. Although workflow tracking takes advantage of user configured dictionaries and protocols, adding additional workflows or steps to existing workflows will require the assistance of a Java software developer.

#### 8)Protocols

**Figure 5:**
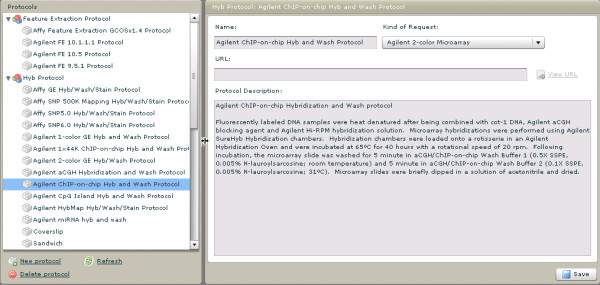
**Protocols**. A generic interface is provided for entering commonly used protocols for both experimental processing and bioinformatic analysis. Shown in these screen captures are protocols for microarray chIP-chip processing.

A catalog of experimental and data analysis protocols is maintained in GNomEx, providing the researcher with summarized descriptions of the methods employed (figure 5). These are used to populate relevant menus and are associated with various steps in sample and raw data processing.

#### 9) Billing

**Figure 6:**
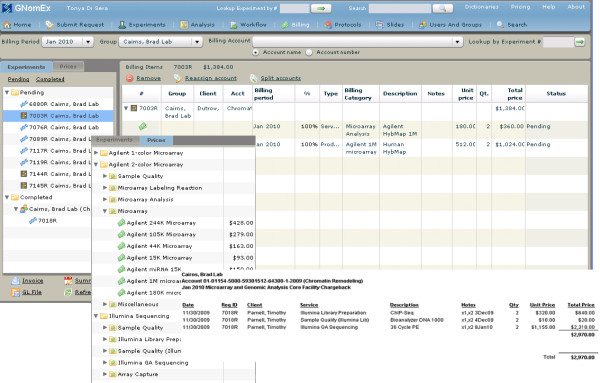
**Billing**. Screen captures of a variety of spreadsheets and forms used to track accounting, billing, and monthly usage reports.

Structured pricing sheets are used to generate itemized charges for each experiment (figure 6). Following the submission of an experiment request, the billing application predicts the required services to fulfill the request by matching experiment details with criteria defined in the end-user configured price sheets. This auto-creation of billing items is performed in Java plug-in modules, which can be recoded by a Java developer to suit the needs of a particular site's billing model. As workflow steps are completed, core facility personnel verify the services and charges and revise or add new billing items where necessary. At month-end, invoices for approved billing items are generated and forwarded to the specified lab billing contact through automated e-mail. A file and report summarizing the charges for each account is prepared for electronic billing of the designated account.

#### 10) Raw data access

**Figure 7:**
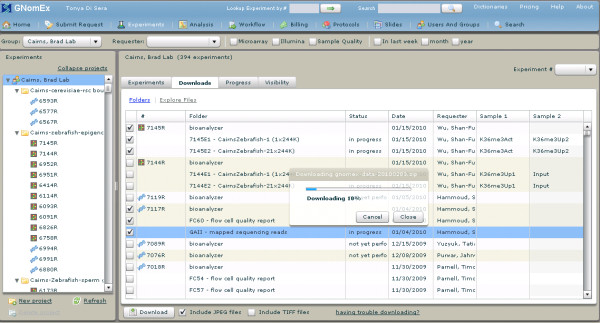
**Raw data download**. Experiment data is manually downloaded using a multiple file selection mechanism. Zip compression is used to speed transfer of data to users. Shown in these screen captures are multiple data selections from different experiments for download.

Raw data files for an experiment request can be downloaded to the researcher's local computer by selecting individual files or entire folders (figure 7). The download is performed through transfer of a compressed zip file and download status is indicated by a progress bar.

#### 11) Processed/analyzed data repository

**Figure 8:**
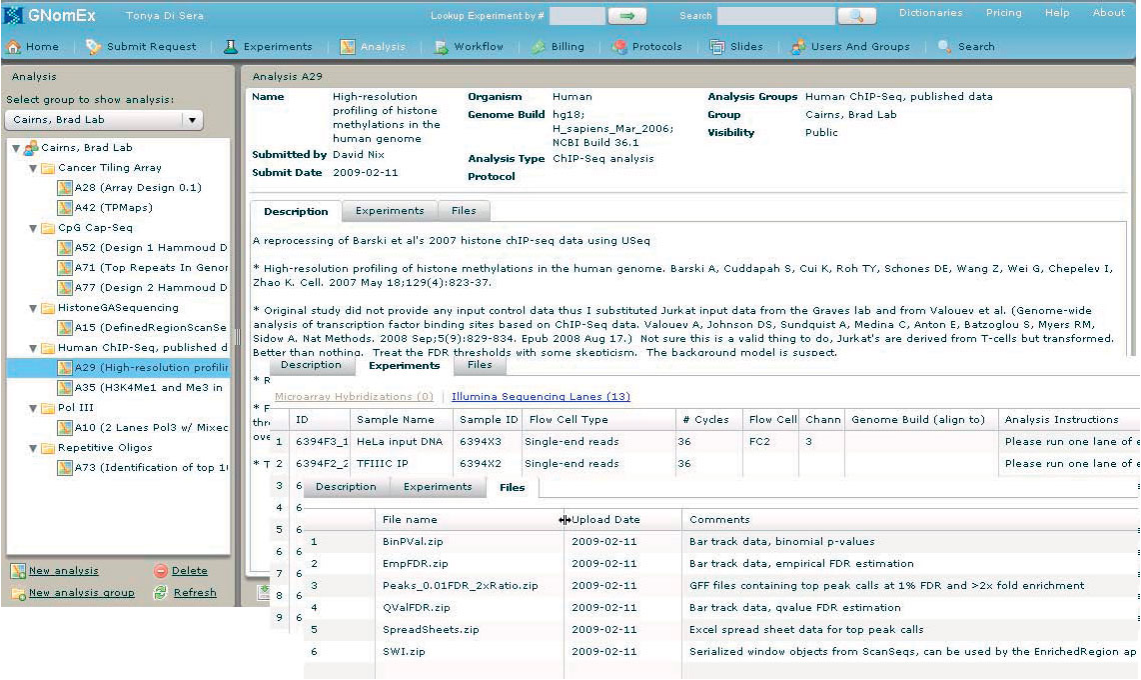
**Processed/analyzed data project center**. In this composite screen capture, the interface for detailing bioinformatic analysis is shown. Multiple analysts can use this project center to keep track of data analysis that has been performed with the same raw datasets, group the analysis into larger projects, and upload analysis files to the repository.

A common problem encountered in working with genomic datasets is managing analyzed data. Too often multiple versions of analyzed data (e.g. gene lists, chip peaks, SNP calls) end up on a user's desktop. Some versions may contain preliminary partial data, some annotated with different genome builds, and some derived using different methods. Keeping track of how the analysis was performed, in what genome build, and using which raw datasets is essential for any subsequent use or replication of the analysis. A key feature of GNomEx is its analysis repository (figure 8). It is designed as a project center where multiple individuals working on the same raw datasets can upload and annotate various aspects of data analysis for large genomic projects. Individual analyses are organized under Analysis groups and annotated for organism, genome build, analysis type, and protocols. Experiments used in the analysis are associated with the analysis through a drag-and-drop mechanism and analyzed data files are uploaded using a multi-file upload widget. The visibility of each analysis is defined by the user.

#### 12) Browsing and Searching

Experiments are organized within project folders that can be browsed according to experiment platform, submission date, or by name of the researcher or lab. Simple text searches as well as advanced, criteria-based searches can be performed on experiments, protocols, and associated analyses. Text searching relies on the high-performance, open source Apache Lucene text search engine [4]. GNomEx keyword searching uses Lucene indexes, built nightly, that contain all text associated with experiments and downstream analysis, including free-form descriptions, structured annotations, sample names, and protocols. Post-search processing culls the results so that only view-permissible data are returned.

## Application: GWrap (Genomic analysis command line tool wrapper)

### Background

Often the best person to analyze genomic data is the person who submitted the samples to the genomics core facility. They typically have an intimate knowledge of the biology behind the project, have a list of key questions to address, and are aware of potentially confounding issues associated with the experiment. Moreover, when they perform their own genomic analysis, they become aware of the various choices made in generating the processed data that limit and bias its contents. As such, a key goal in our bioinformatics shared resource is to enable users to analyze their own data. For some genomic datasets users can choose from a variety of mature, open access, user friendly, GUI based applications for data processing. (e.g. gene expression, SNP genotyping). For other more recently emerging datasets, such as those derived from tiling microarray and next generation sequencing platforms, sophisticated well characterized analysis tools do exist but are often challenging to use given their command line interface. This is to be expected. Analysis software evolves from minimalistic command line scripts, to integrated command line packaged tools, to web and stand alone GUI applications. When novel analysis approaches change frequently, designing and updating GUIs is often viewed as unproductive by application developers. On the other hand, many scientists avoid command line programming. To break this impasse, web based wrapper applications such as Galaxy [5] and GenePattern [6] have proven useful. Users upload their data to a remote server, use web forms to execute command line applications, and download their analysis all in the framework of a web browser. Although effective, it can be less than ideal for processing large tiling microarray and next generation sequencing datasets. The gigabyte size of these datasets poses problems for timely data upload and download, for data storage on a central server, and requires extensive computational resources to process one dataset, let alone multiple datasets from multiple users. Lastly, from a developer standpoint, creating the web forms for each command line application and keeping them up to date requires effort that is often better spent improving the underlying algorithms.

### Implementation and Results

**Figure 9:**
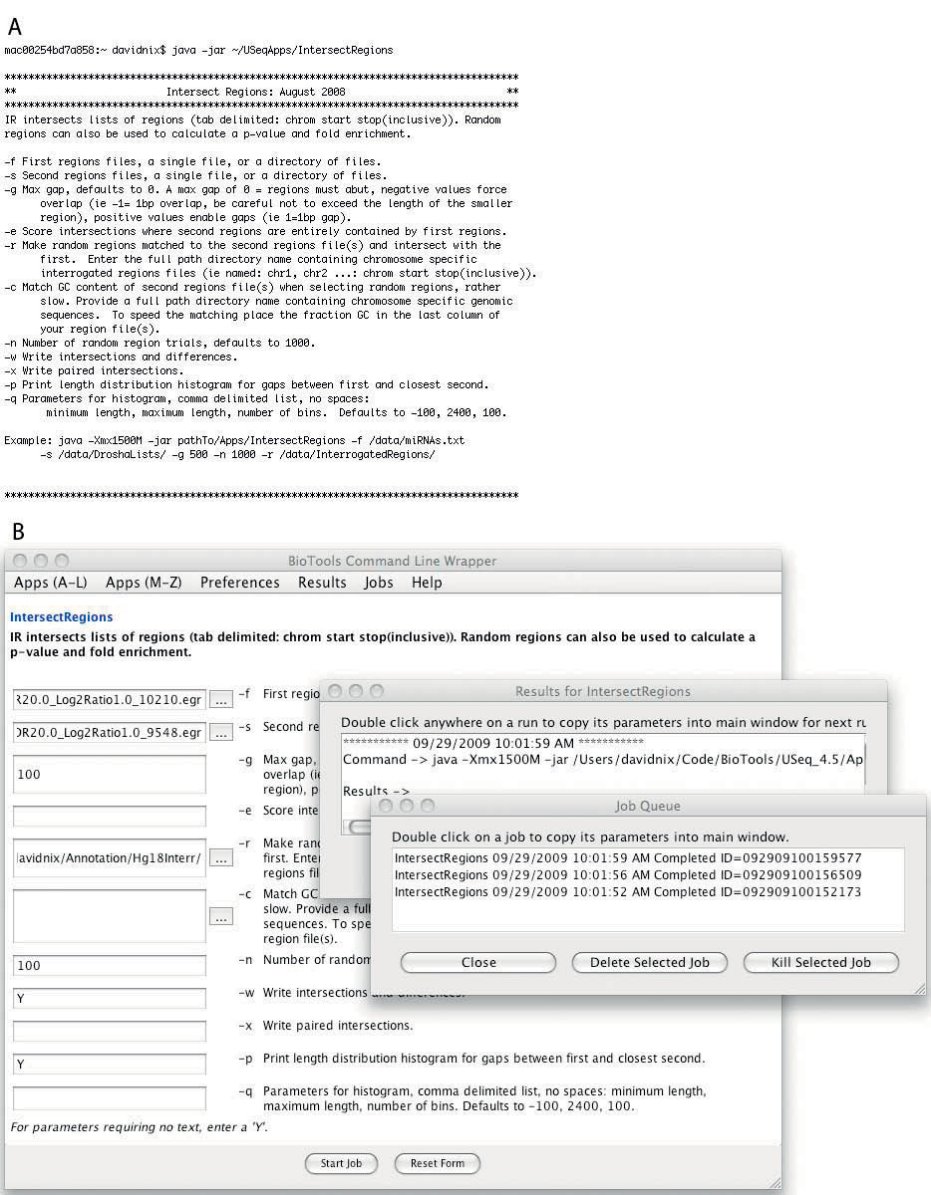
**GWrap**. Shown in these screen captures is the conversion of a command line application (A) to a GUI (B) using the stand-alone Java swing GWrap application.

We have taken an alternative approach that uses a stand-alone Java Swing application to wrap command line tools into a user friendly GUI (GWrap, figure 9). Upon launch GWrap executes each application in the "Apps" directory, parses their menus, and generates application specific forms. HTML files in the "Documentation" directory are added as links to GWrap's help menu. Jobs are launched by completing a GWrapped form or by populating it with a prior run history. Jobs are added to a queue and launched sequentially. Standard out and error streams are associated with the run parameters and archived. The advantages of this approach are numerous. Using GWrap, 120 command line applications from our USeq [7,8] next generation sequencing and TiMAT [9] tiling microarray analysis packages can now be run in a user friendly, point and click, file drag and drop GUI. GWrap, USeq, and TiMAT2 are platform independent and run on any operating system that supports Java. By running on the user's computer, there are no file upload or download issues, nor problems associated with overloading of computational resources by external users. Lastly, changes to the command line applications are automatically incorporated into the GUI. Although GWrap is configured to work with Java JAR applications and parse a standardized command line menu (see the cmdLnMenus.html file in the USeq package [7] for some examples), it can be modified to work with command line scripts written in any language. GWrap allows investigators, who prefer GUIs over command line programs, to run their own analysis using cutting edge computational analysis applications without burdening developers with additional GUI development.

## Application: GenoPub (Genomic Annotation Publisher)

### Background

Another key issue associated with effective use of genomic experiments in laboratories and clinics is the difficulty in efficiently distributing analyzed data. Too often, analyzed data are placed in a supplemental data folder on an author's or journal's web site where annotation of the analysis is non-standard and typically incomplete. Determining which methods were used in generating the data, or even the genome build, is often difficult. Submission of analyzed data to a public repository such as GEO [10] or ArrayExpress [11] is an improvement but is rarely done except when publishing the original unprocessed data. Some bioinformatic groups such as UCSC Genome Bioinformatics [12,13] will host external datasets provided one can convince them it is of interest to their users. In all cases, the data cannot be integrated in a subsequent analysis without extensive manual file downloading, filtering, and reformatting. Making a simple visual comparison between different datasets from different data sources in a genome browser requires considerable effort. Hundreds of genomic datasets are currently buried in web archives or customized databases. As such they are effectively inaccessible. Ideally, a researcher would distribute their own data on the internet using a common protocol so that other groups could see it and could *programmatically *download portions of it for subsequent comparison with other datasets.

A solution to this problem exists and has been in development for more than ten years. It makes use of a Distributed Annotation System (DAS) protocol, and a DAS server [14-19]. DAS is a communication protocol developed to exchange annotations on genomic and protein sequences between servers and client applications over the internet. Hundreds of DAS/1 servers are in use at bioinformatic data centers such as WormBase, UCSC, Ensembl, FlyBase, TIGR, and UniProt. Unfortunately, the DAS/1 protocol is not amenable for distributing large genomic datasets given its requirement that datasets be formatted using verbose text based DAS XML. DAS/2 [20] is a recent extension of the DAS/1 protocol and is optimized for distributing large genomic datasets in both text and binary formats (e.g. bed, gff3, wig, bar, fasta, useq, dasXML, sam, bam). The difference in file size and corresponding download time between gzip compressed DAS XML and a binary format like useq is typically >100 fold (e.g. 85 MB vs 0.6 MB for the ENCODE's wgEncodeBroadChipSeqSignalGm12878Ctcf chIP-seq graph data for chr21). Any dataset that can be associated with a specific genome build and genome coordinates (e.g. gene expression, SNP, CNV, chIP-chip, chIP-seq, RNA-seq, chromosomal rearrangements) can be efficiently shared between DAS/2 servers and DAS/2-enabled clients such as IGB [1] and GBrowse [21] or incorporated into data objects from the Cancer Biomedical Informatics Grid (caBIG).

### Implementation and Results

We have adopted DAS as our genomic data distribution model and have been working with the GenoViz open source project [1,19,22] to extend the functionality of the GenoViz Genometry DAS/2 server in three key areas. The first improvement was to implement a user-group public-private security model using http md5 digest authentication to enable restricted access of designated genomic datasets to particular users. Researchers need to be able to compare their unpublished data with public datasets. Clinicians working with patient data require controlled access under all situations. If needed, these servers can leverage other internet based security protocols such as secure socket layers and virtual private networks used by banks and hospitals for securing internet data exchange.

A second improvement was to develop a compressed, pre-indexed, binary data format called useq, that would support the majority of high throughput genomic text based data formats (e.g. bed, gff, gtf, wig, sgr, gr) in a manner that would not require indexing upon server start up nor loading of the data into memory. The GenoViz DAS/2 server was built using an in memory data distribution model. This is appropriate for reference annotations and enables a rapid response to DAS/2 requests. The useq data format provides a mechanism for hosting a large number of high-density datasets limited only by disk space. Tools for generating and extracting information from useq archives are distributed with the USeq package. A detailed description of the format is included in the USeq documentation [23].

**Figure 10:**
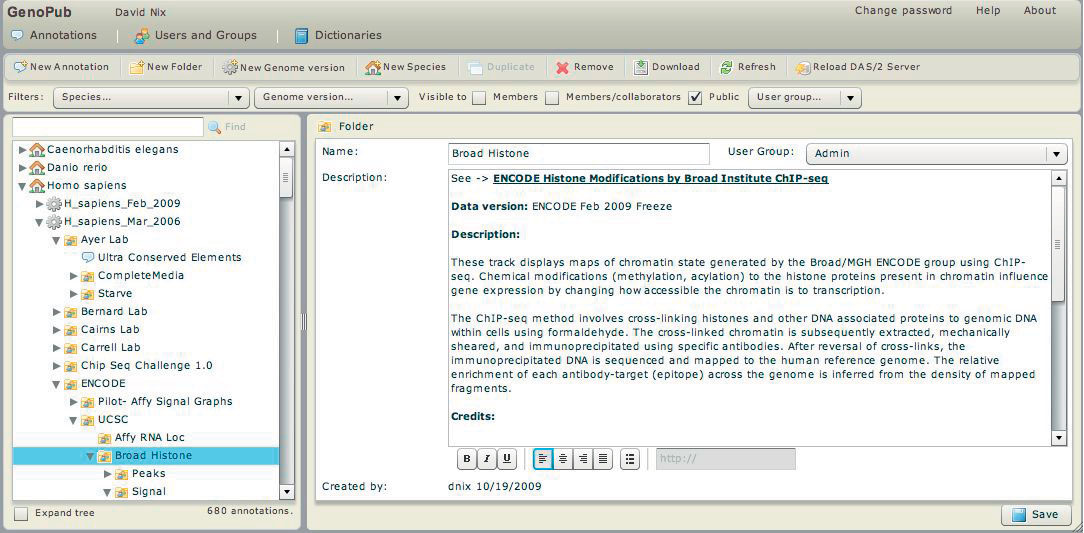
**GenoPub**. This screen capture shows the main annotation window of GenoPub where users organized data for DAS/2 distribution under particular genome builds and data folders. A combination of free text and restricted dictionary driven data fields are provided to describe the user's data.

The third improvement was to create a user-friendly GUI based web application called GenoPub (figure 10) to organize and annotate the genomic datasets distributed by the GenoViz DAS/2 server. GenoPub is a front end to the GenoViz DAS/2 server. It uses a relational database to associate meta data such as author, experiment platform, experimental method, analysis type, and a free text description with genomic datasets grouped by species and genome build. Files and directories of annotation files are added to a particular build using a drag-and-drop mechanism. This meta data is included in the XML DAS/2 types response as property key-values for subsequent DAS client application display. GenoPub allows the same data to be organized into multiple views. As with GNomEx, GenoPub uses the rich client Flex interface that runs in Flash within a user's web browser to interact with the Java classes and a relational database. It is built as a stand-alone application independent of GNomEx and can be deployed using open access relational databases and servlet containers (e.g. MySQL and Apache Tomcat). Although the installation of GenoPub and its dependencies requires some computer literacy, GenoPub is designed to be used and administered by researchers with minimal computer skills. It is our hope that groups generating genomic datasets will distribute their data using DAS/2 alongside their group or institutional web site. To publish their data they simply register their DAS/2 URL with the BioDAS registry [24] and include it as a web link on their laboratory web site.

**Figure 11:**
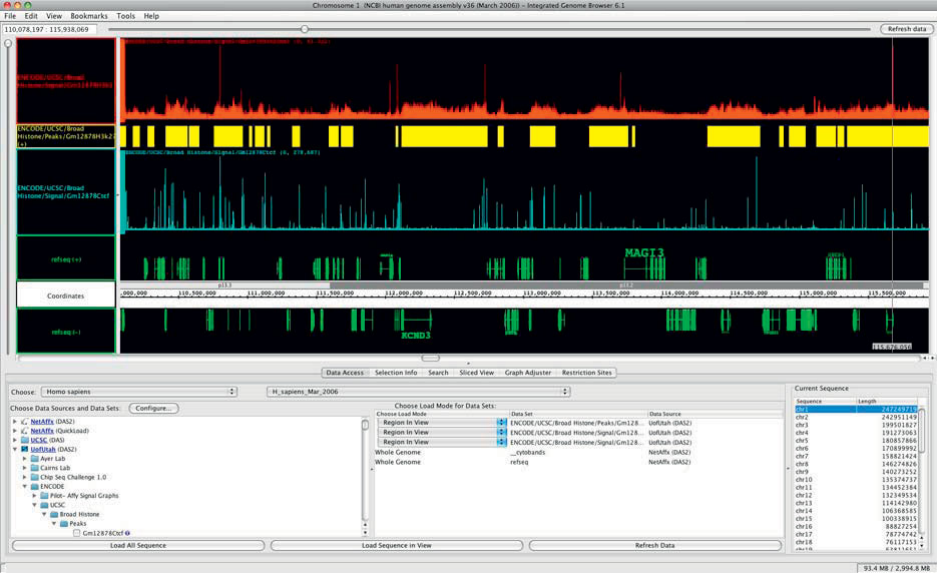
**A DAS/2 client application, the Integrated Genome Browser**. Shown here is a screen capture of IGB's main track browser window and its Data Access tab. The latter is used to select resources to load from a variety of remote servers including QuickLoad, DAS/1, and DAS/2 data sources. A combination of track data from the Broad's chIP-seq ENCODE project (red and blue), peak calls (yellow), and gene annotation (green) are displayed.

The GenoPub web application provides an efficient, easy to use tool for organizing, annotating, and distributing genomic data using the community vetted DAS/2 protocol. Although data can be retrieved manually using DAS queries, programmatic access through DAS/2-enabled client applications such as IGB are ideal. IGB is a sophisticated, open source, cross platform, stand alone genome browser designed for real time visualization and manipulation of large genomic datasets (e.g. chIP-seq, RNA-seq, transcriptome, copy number, SNP/INDEL, BAM alignments, gene expression, tiling microarray, etc) [1,19,22]. We have worked extensively with the GenoViz code and the BioViz group [25] at the University of North Carolina at Charlotte to better integrate DAS/2 queries and response into IGB (figure 11). Currently, 25 laboratories at the University of Utah are using GenoPub and IGB to distribute and visualize ~1100 genomic datasets. We are also publically hosting ~600 chIP-chip, chIP-seq, and transcriptome datasets from a variety of sources including signal and chIP-seq peak calls from the UCSC ENCODE project and several large chIP-seq mapping projects [26-28]. Although DAS/2 and IGB provide a well-developed genomic data distribution and visualization model, access to genomic data should not be tied to any particular analysis or visualization application. We strongly encourage software developers to include support for DAS/2 queries in their genomic applications. Public data repositories such as GEO, NCBI, ArrayExpress, and other large genomic institutions would do the research community a great service by providing unfettered programmatic access to genomic datasets using a common communication protocol such as DAS/2.

## Conclusions

Presented here are three software applications developed to assist with generating, annotating, analyzing, organizing, distributing, and visualizing genomic data. GNomEx is the first published open source genomic LIMS that supports next generation sequencing and microarray platforms. It is an enterprise level application built for integrating multiple university core facilities and dovetails with the Bio Sample Tracking database in use at the University of Utah and Huntsman Cancer hospitals. Unlike most other LIMS, GNomEx contains an analysis project center where multiple users can upload, annotate, and associate analysis with the raw data archived in GNomEx. This is a critical feature needed to maintain a chain of custody type tracking of patients to samples to raw data to analyzed data. To efficiently distribute this processed data, we developed an easy to use web application called GenoPub. GenoPub associates and distributes meta data with each analyzed dataset through the GenoViz DAS/2 server. Analysis can be organized under multiple views (e.g. by patient, disease, or factor) and restricted to particular users enabling the controlled distribution of patient and unpublished data alongside public datasets. To obtain analysis, users either manually download it to their local computer or access it programmatically through DAS/2-enabled client applications such as IGB.

These tools provide critical infrastructure for efficiently managing and distributing genomic data for use in the laboratory and the clinic and return the focus of genomic bioinformatics to data analysis. The development of novel analysis methods is accelerating as fast as next generation sequencing costs fall. Unfortunately, making these cutting edge analysis tools accessible to a wide spectrum of users is proving difficult. One solution presented here makes use of a stand alone GUI, GWrap, to convert 120 command line applications found in two widely used next generation sequencing and tiling microarray analysis packages, USeq and TiMAT2, into a user friendly GUI without placing a burden on developers nor compromising the command line interface. GWrap can be incorporated into other analysis packages with minimal effort. In summary, we believe these next generation tools are well suited for making the best use of datasets from the post-genomic era.

## Availability and requirements

Project names: GNomEx, GWrap, GenoPub

Project home pages: http://sourceforge.net/projects/gnomex, http://sourceforge.net/projects/useq, http://sourceforge.net/projects/genoviz

Operating systems: Platform independent

Programming languages: Java

Other requirements: Java 1.6+, a relational database (e.g. MySQL, Microsoft SQL Server), object/relational database mapping tool Hibernate 3.2+ https://www.hibernate.org, a Java servlet container (e.g. Apache Tomcat, Orion)

Licenses: GPLv3 for GNomEx, BSD for GWrap and USeq, Common Public License for GenoPub

Restrictions: For profit organizations are required to obtain a commercial license before deploying GNomEx in whole or part. No such restrictions are in place for USeq, GWrap, or GenoPub. See the licence.txt document in the individual package downloads for details.

## Abbreviations

**LIMS**: Laboratory Information Management System; **GUI**: Graphical User Interface; **IGB**: Integrated Genome Browser; **DAS**: Distributed Annotation System; **MGED**: Microarray Gene Expression Databases; **SNP**: single nucleotide polymorphism

## Authors' contributions

TLD designed and wrote essentially all of the software for GNomEx and GenoPub with minor contributions from DAN. The specifications for the software were developed by DAN, TLD, BKD, BAM, and SJC. DAN and RMC designed and wrote the GWrap application. DAN, TLD, and KSQ contributed software code to extend the functionality of the GenoViz DAS/2 server and IGB applications. Significant portions of GNomEx were designed using a prior LIMS system (ArrayInfo) developed by BAM. DAN wrote the manuscript. SJC managed the entire project. All authors have read and approved this manuscript.
